# Serum IGF-1 Affects Skeletal Acquisition in a Temporal and Compartment-Specific Manner

**DOI:** 10.1371/journal.pone.0014762

**Published:** 2011-03-18

**Authors:** Hayden-William Courtland, Sebastien Elis, Yingjie Wu, Hui Sun, Clifford J. Rosen, Karl J. Jepsen, Shoshana Yakar

**Affiliations:** 1 Division of Endocrinology, Diabetes and Bone Disease, Mount Sinai School of Medicine, New York, New York, United States of America; 2 Leni and Peter W. May Department of Orthopaedics, Mount Sinai School of Medicine, New York, New York, United States of America; 3 Maine Medical Center Research Institute, Scarborough, Maine, United States of America; Buck Institute for Age Research, United States of America

## Abstract

Insulin-like growth factor-1 (IGF-1) plays a critical role in the development of the growing skeleton by establishing both longitudinal and transverse bone accrual. IGF-1 has also been implicated in the maintenance of bone mass during late adulthood and aging, as decreases in serum IGF-1 levels appear to correlate with decreases in bone mineral density (BMD). Although informative, mouse models to date have been unable to separate the temporal effects of IGF-1 depletion on skeletal development. To address this problem, we performed a skeletal characterization of the inducible LID mouse (iLID), in which serum IGF-1 levels are depleted at selected ages. We found that depletion of serum IGF-1 in male iLID mice prior to adulthood (4 weeks) decreased trabecular bone architecture and significantly reduced transverse cortical bone properties (Ct.Ar, Ct.Th) by 16 weeks (adulthood). Likewise, depletion of serum IGF-1 in iLID males at 8 weeks of age, resulted in significantly reduced transverse cortical bone properties (Ct.Ar, Ct.Th) by 32 weeks (late adulthood), but had no effect on trabecular bone architecture. In contrast, depletion of serum IGF-1 after peak bone acquisition (at 16 weeks) resulted in enhancement of trabecular bone architecture, but no significant changes in cortical bone properties by 32 weeks as compared to controls. These results indicate that while serum IGF-1 is essential for bone accrual during the postnatal growth phase, depletion of IGF-1 after peak bone acquisition (16 weeks) is compartment-specific and does not have a detrimental effect on cortical bone mass in the older adult mouse.

## Introduction

Hormonal regulation throughout growth and adulthood is known to affect skeletal strength. The interplay between hormones and genetic factors accounts for significant variability in adult BMD, bone quality, and the kinetics of bone accrual during growth and bone loss during aging. IGF-1, which acts in an endocrine and autocrine/paracrine manner, is one hormone implicated in the establishment and maintenance of bone integrity. At the cellular level, numerous *in vitro* and *in vivo* studies demonstrated that IGF-1 can affect osteoblast growth and differentiation [1], induce type I collagen expression [2] and inhibit type I collagen degradation in differentiated fetal rat osteoblasts [3], [4]. Further, IGF-1 plays an important role in cell survival [5], [6], promotes the formation of osteoclast-like cells from mononuclear precursors and can subsequently stimulate the resorptive activity of osteoclasts [7], [8]. Thus, IGF-1 plays a fundamental role in coordinating the cellular processes of bone formation (apposition) and bone removal (resorption).

Serum IGF-1, which is produced largely by the liver, contributes to the overall skeletal pool of IGF-1. During puberty, in both humans and mice, linear growth and bone formation are associated with a rise in serum and skeletal IGF-1. In mice, a rapid body weight gain occurs from birth up to 4 weeks of age [9], [10]. During this period, liver IGF-1 gene expression is activated and rises to adult levels [11]. Somatic growth during this early growth phase is affected by global IGF-1 gene deficiency. For example, IGF-1 null mice exhibit a 35% growth retardation at birth, which increases to 60% by 3 weeks of age [12]. Thus, IGF-1 is crucial in promoting bone growth during the early growth phase (1–28 days). Early bone growth plays a critical role in defining adult skeletal integrity because during this time ∼80–90% of adult femoral total area (Tt.Ar) is established [9], but the later growth phase (4–16 weeks) is also important as gains in the amount of cortical bone tissue area (Ct.Ar) increase significantly through the addition of bone on both the outer (perisoteal) and inner (endosteal) surfaces [9], and small gains in longitudinal bone growth exists since the femoral growth plate does not close after sexual maturation in mice as it does in humans. These ontogenic changes in bone size and amount are clearly dependent on serum IGF-1 levels as liver IGF-1 deficient (LID) mice, with 75% reductions in serum IGF-1 from birth, exhibit only ∼6% decreases in bone length but significant reductions (∼30%) in bone mineral density (BMD), Tt.Ar, and Ct.Ar by the middle of the late growth phase (8 weeks) [15].

In contrast to our understanding of growth and development, the contribution of serum IGF-1 to skeletal integrity during adulthood remains unclear. In order to clarify the relationship between serum IGF-1 levels and skeletal structure in adulthood (after 16 weeks), the temporal variation in serum IGF-1 must be specifically addressed. Given that our LID mouse model exhibits a constitutive serum IGF-1 deficiency (from birth) we could not rule out an accumulating effect of IGF-1 on bone development in these animals. To quantify the effects of IGF-1 during defined growth phases, we developed an inducible liver IGF-1 deficient mouse model (iLID) that allows us to deplete circulating IGF-1 at specific stages of mouse development. The iLID model is based on the Cre/loxP system whereby the Cre recombinase is expressed specifically in the liver under the anti-trypsin 1α promoter, and can be induced by a single tamoxifen injection that does not otherwise affect the skeleton. This model permits us to dissect the temporal contribution of circulating IGF-1 and contrast the early and later effects of IGF-1 on skeletal acquisition, maintenance, and micro-architecture.

## Materials and Methods

### Animals

The iLID model (C57BL/6 backgruond) has been described previously [16]. Briefly, iLID was created using the Cre-loxP system and crossing mice with floxed exon 4 of the IGF-1 gene with transgenic mice expressing tamoxifen-inducible Cre-recombinase under the trypsin-1a promoter. In this model, iLID mice (which are homozygous for the floxed IGF-1 and carry the Cre-recombinase transgene) exhibit normal levels of IGF-1 in serum. Upon tamoxifen injection, an igf-1 gene recombination occurs specifically in hepatocytes, leading to ∼60% reductions in serum IGF-1 levels. Control mice are homozygous for the floxed IGF-1 allele but do not carry the Cre-recombinase and therefore do not recombine the igf-1 gene in response to tamoxifen injection. Male mice used in this study were given unrestricted access to water and food, and housed to a maximum of 5 per cage under a 12 hours light:dark cycle. Animal care and maintenance were provided through the Mount Sinai School of Medicine AAALAC Accredited Animal Facility. All procedures were approved by the Institutional Animal Care and Use Committee of the Mount Sinai School of Medicine (protocol number 06-1061).

### Validation of the iLID Model

As this investigation required the use of tamoxifen to induce Cre-recombinase-mediated gene recombination in liver, we needed to determine the minimal dose of tamoxifen that would 1) reduce serum IGF-1 levels by at least 50% in the iLID mice without affecting bone remodeling *per se* and 2) not affect circulating IGF-1 levels in *floxed* controls. The first part of this validation has been published previously. Tamoxifen (Sigma Aldrich, St. Louis, MO, USA) was dissolved in seed oil with 10% ethanol at a concentration of 3 mg/ml, sonicated for 30 minutes and injected intraperitoneally to both iLID and control mice at the indicated time points. We examined a dose response effect of tamoxifen on serum IGF-1 levels as well as on *igf-1* gene expression in liver [16]. The minimal effective dose of tamoxifen in the adult mouse was a single injection of 0.3 mg of tamoxifen. Using this dose we were able to decrease serum IGF-1 by 60% in the iLID compared to vehicle treated control (seed oil). We also verified the time course of serum IGF-1 decrease and revealed that at ∼18 hours after tamoxifen injection both serum IGF-1 levels and liver IGF-1 mRNA levels were decreased [16].

### Serum Hormones

Serum was obtained from blood samples after mandibular bleeding in the fed state. Serum levels of IGF-1 and GH were determined using commercial radio-immuno assays as described previously [15], [17], [18].

### Bone Morphology and Microarchitecture

Diaphyseal and distal metaphyseal regions of left femora were analyzed using micro-computed tomography (micro-CT). A three-dimensional micro-CT image of the entire femur was obtained using an eXplore Locus SP micro-CT system (GE Healthcare, London, Ontario, CA), which was engineered to minimize beam-hardening and to produce field flatness through the use of x-ray filters and an equilibration bath. Scans were performed at 8.7 µm voxel resolution and bone was delineated from non-bone using a standard global thresholding algorithm. From the reconstructed micro-CT images, a 2 mm region of the mid-diaphysis (cortical bone) beginning immediately distal to the third trochanter was examined for total cross-sectional bone area (Tt.Ar), cross-sectional marrow area (Ma.Ar), cortical bone (tissue) area (Ct.Ar), polar moment of inertia (J_o_), cortical thickness (Ct.Th). Relative cortical area (RCA) was defined as Ct.Ar/Tt.Ar and represented the relative amount of bone tissue packed into a give bone area. From the reconstructed images of the distal metaphysis, a 2 mm region (trabecular bone) immediately proximal to the growth plate was examined for bone mineral density (BMD), bone volume/total volume (BV/TV), trabecular number (Tb.N), trabecular thickness (Tb.Th), and trabecular spacing (Tb.Sp). BMD was calculated as the ratio of bone mineral content (BMC, mg) to the total volume of distal metaphysis analyzed after removing (masking) marrow space grayscale variability. In addition, tissue mineral density (TMD) values were obtained which represent the amount of mineral in a given bone tissue as compared to a hydroxyapatite standard.

### Histology

Animals were injected with calcein (20 mg/kg) twice before sacrifice. Isolated femora were cleaned of soft tissue, fixed in 10% buffered formalin and embedded in poly-methylmethacrylate (PMMA). For trabecular analysis, longitudinal sections (5 µm thick) were cut at the 50% coronal plane using a Leica 2265 microtome, deplasticized and stained with Goldner's Trichrome stain for static measurements based on morphology (e.g., osteoblast and osteoclast number). Additional sections were cut at 10 µm for the dynamic (fluorescent) measurements. For each sample, a region of interest was defined 250 µm distal to the growth plate and extending 1 mm downward (thereby avoiding the primary spongiosa) through the metaphysis of the femur. Standard bone histomorphometry was performed as described previously [19] using Bioquant Image Analysis software (R & M Biometrics, Nashville, TN). For cortical analysis, 200 µm sections were cut at the mid-diaphysis using a low-speed diamond saw (Buehler, Inc., Lake Bluff, IL, USA). Sections were mounted on glass sides, polished to ∼50 µm, and analyzed for dynamic (fluorescent) measurements using OsteoMeasure (Osteometrics, Atlanta, GA, USA) as described previously [19].

### Gene Expression

Total RNA was extracted from liver samples using TRIzol reagent according to the manufacturer's instructions (Invitrogen Corp., Carlsbad, California, USA). RNA integrity was verified using Bioanalyzer (Agilent Technologies 2100 Bioanalyzer-Bio Sizing, Version A.02.12 SI292). One µg of RNA was reverse-transcribed to cDNA using oligo(dT) primers with a RT-PCR kit according to the manufacturer's instructions (Invitrogen). Quantitative RT-PCR was performed with the QuantiTect^TM^ SYBR^®^ green PCR kit (Qiagen, Valencia, CA, USA) according to the manufacturer's instructions in ABI PRISM 7900HT sequence detection systems (Applied Biosystems, Foster City, CA USA). Each transcript in each sample was assayed three times and the fold change ratios between experimental and control samples were calculated relative to β-actin.

### Statistics

Statistical differences between mean trait values of iLID mice and control mice were assessed using one-way ANOVA and Bonferroni post-hoc tests (p<0.05, Statistica 6.0, Statsoft, Tulsa, OK, USA).

## Results

### Validation of the iLID model for Skeletal Studies

**Figure 1:**
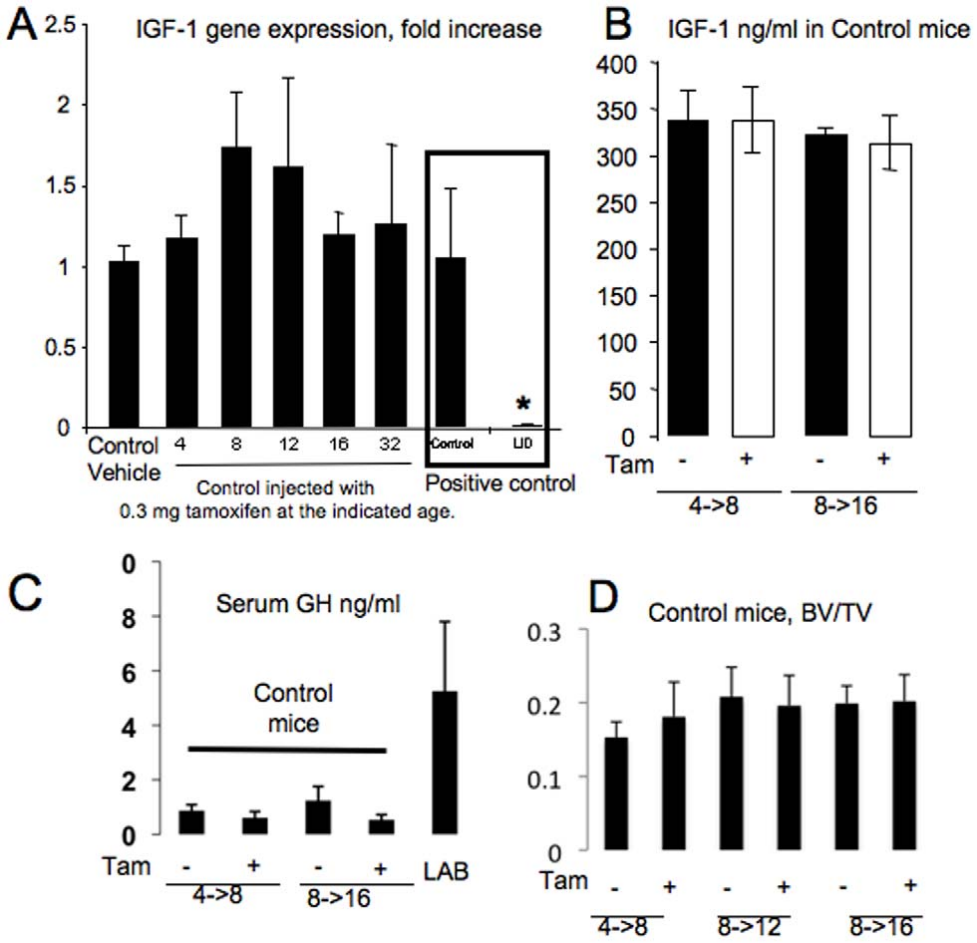
Validation of the iLID model. (A) Liver expression of IGF-1 in *control* mice (mean ± SE) is not altered following 0.3 mg tamoxifen injection. *Control* mice at 4, 8, 12, 16 and 32 weeks of age were injected with 0.3 mg tamoxifen and livers were dissected four weeks later for gene expression analysis using real time PCR (*n* = 3 per group). Control and “constitutive” LID mice were used as positive control for the assay. Cycle number was corrected to β-actin and levels related to vehicle control, which was set as 1. (B) Mean serum IGF-1 levels in *control* mice (± SE) injected with 0.3 mg of tamoxifen (+) at 4 or 8 weeks were not altered compared to vehicle treated controls (−) at 8 or 16 weeks, respectively. (C) Mean serum GH levels in *control* mice (± SE) injected with 0.3 mg of tamoxifen (+) at 4 or 8 weeks were not altered compared to vehicle treated controls (−) at 8 or 16 weeks, respectively. Sera from triple knockout of LID/acid labile subunit KO/IGFBP-3KO (LAB) mice, which have high serum GH levels [24], served as a positive control. (D) BV/TV (bone volume/total volume) from micro-CT analysis of *control* mice injected with vehicle (−) or 0.3 mg tamoxifen (+) at 4, 8, and 12 weeks of age and then analyzed at 8, 12, and 16 weeks, respectively. Trabecular bone was analyzed at the femoral distal metaphysis (*n* = 6 per group). No significant differences were found between vehicle and 0.3 mg tamoxifen injected mice.

To address whether injection of 0.3 mg of tamoxifen affected liver igf-1 gene expression in wild-type mice, we injected 0.3 mg of tamoxifen into control mice at different ages and assessed liver IGF-1 mRNA levels by real time PCR. No differences in *igf-1* gene expression were detected between tamoxifen and vehicle-injected control mice at any age (4, 8, 12, 20 and 32 weeks) (Figure 1A). Serum IGF-1 levels following a 0.3 mg tamoxifen injection were evaluated using radio-immunoassay and revealed no differences between tamoxifen and vehicle injected control mice (Figure 1B). In the LID model we showed that the low levels of serum IGF-1 were not adequate to inhibit pituitary GH secretion. As seen in Figure 1C, serum GH levels were not affected by 0.3 mg tamoxifen injection in control mice. To quantify the effects of 0.3 mg of tamoxifen on the trabecular architecture, we analyzed 33 femurs of male control mice at different ages. Our results revealed no differences in distal femoral BV/TV in control mice injected once with tamoxifen at 4 weeks and then analyzed at 8 weeks, nor any differences in animals injected at 8 weeks and analyzed at 12 or 16 weeks (Figure 1D).

### Depletion of serum IGF-1 levels during puberty leads to reductions in cortical and trabecular bone acquisition by adulthood

**Figure 2:**
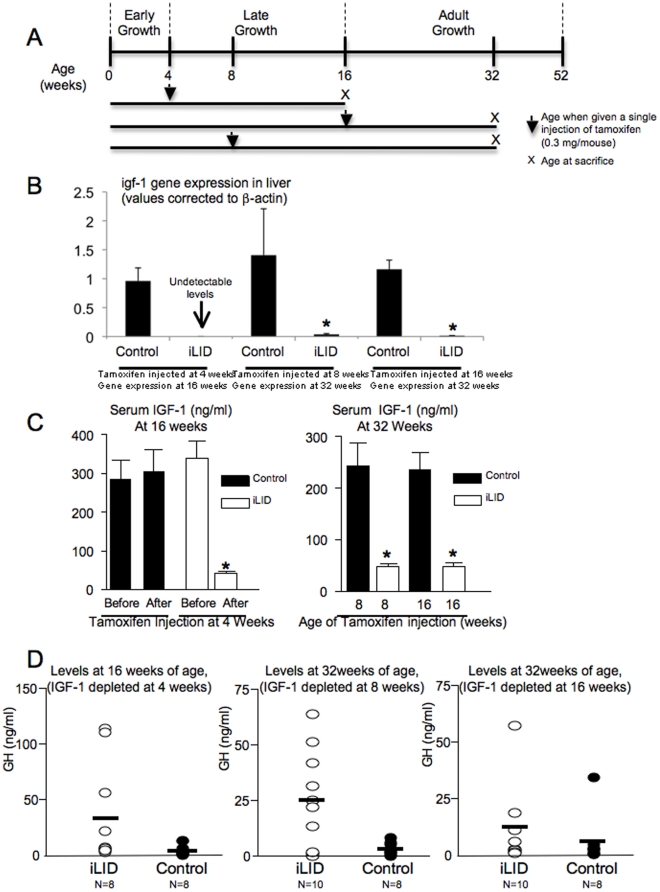
Experimental design, gene expression and serum levels for control and iLID mice. A) Schematic representation of the experimental design. (B) igf-1 gene expression in liver in control and iLID mice injected with tamoxifen at different ages (C) Mean serum IGF-1 levels (± s.d) in control (*n* = 6) and iLID (*n* = 6 each) before (4 weeks) and after (16 weeks) injection of 0.3 mg of tamoxifen at 4 weeks. Serum IGF-1 levels in iLID mice are statistically different from control mice as expected (ANOVA, p<0.05). (D) Serum GH levels in control (*n* = 8) and iLID mic (*n* = 10) after depletion of serum IGF-1 by tamoxifen injection.

As a result of the aforementioned experiments, a single 0.3 mg/mouse injection of tamoxifen was chosen as an appropriate dose to study the effect of temporal IGF-1 deletion on the skeletal development of growing mice. Using the iLID model only one injection of tamoxifen is required since hepatocytes (in which gene recombination is induced) have low turnover rate and the genetic recombination is persistent for at least 16 weeks. Our experimental design is presented in figure 2A; male control and iLID mice were injected once with tamoxifen (0.3 mg/mouse) at 4, 8 or 16 weeks of age and were followed until peak bone acquisition, at 16 (based on prior studies of cortical bone growth in C57BL/6 mice [9], [13]) or at 32 weeks of age. A single injection of tamoxifen resulted in decreased igf-1 gene expression in liver of all iLID groups (Figure 2B) and consequent reductions in serum IGF-1 levels, which persisted until sacrifice at 16 or 32 weeks of age in iLID mice (Figure 2C). The reduction in serum IGF-1 levels in the iLID mice were accompanied by elevations in serum GH levels, which were detectable at the age of sacrifice (Figure 2D).

**Figure 3:**
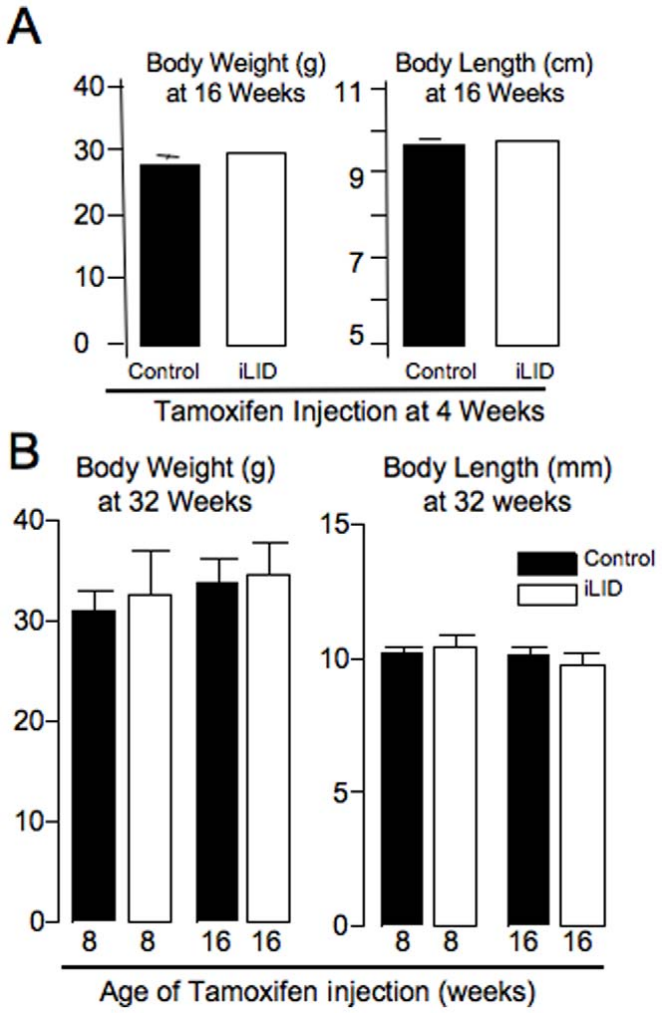
Body weight and length data for control and iLID mice. (A) Mean body weight and body length (± SE) of control (*n* = 6) and iLID (*n* = 6) mice at 16 weeks after injection of 0.3 mg of tamoxifen at 4 weeks. (B) Body weights and body lengths (± s.d) for 32 week control and iLID after injection of tamoxifen at either 8 or 16 weeks of age.

**Figure 4:**
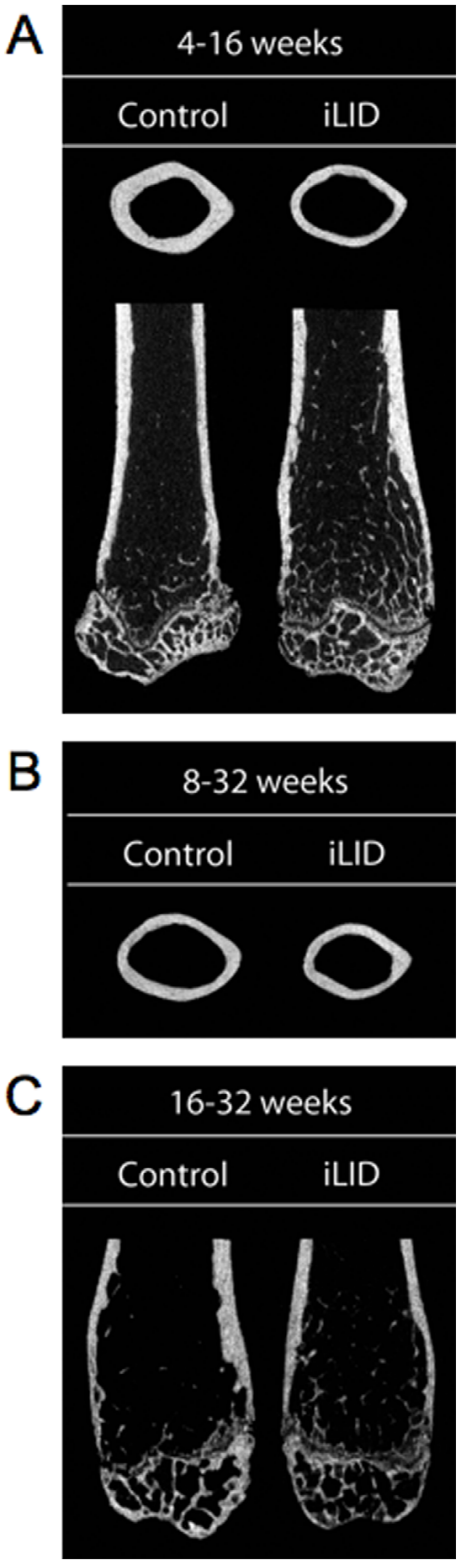
Micro-CT images of control and iLID mice indicating differences in cortical and trabecular bone accrual. (A) Mice injected with tamoxifen at 4 weeks and sacrificed at 16 weeks. (B) Mice injected with tamoxifen at 8 weeks and sacrificed at 32 weeks. (C) Mice injected with tamoxifen at 16 weeks and sacrificed at 32 weeks.

When depleted of serum IGF-1 at 4 weeks, body weight and body length of iLID mice did not differ significantly from controls at 16 weeks of age (Figure 3A). However, 16-week cortical bone, analyzed at the mid-diaphysis, showed significant reductions in Ct.Ar and Ct.Th in iLID mice depleted of serum IGF-1 at 4 weeks of age (Table 1, Figure 4A). Although histomorphometric analysis of cortical bone did not show significant differences between control and iLID mice at this age, likely due to the fact that bone formation slows appreciably by 16 weeks, mean values were all lower for iLID mice as compared to controls (Table 2). Trabecular architecture from the femoral distal metaphysis was also quantified using micro-CT (Table 1, Figure 4A). We found that depletion of serum IGF-1 beginning at 4 weeks of age resulted in a significant increase in Tb.N by 16 weeks of age compared to the vehicle treated group. There was also a concomitant decrease in Tb.Th and Tb.Sp for both groups.

**Table 1:** Mean cortical and trabecular bone traits values (± s.d) obtained from micro-CT measurements of male femora from control (*n* = 6) and iLID mice (*n* = 6) at 16 weeks of age after injection with tamoxifen at 4 weeks.

Cortical Bone Traits	Control 4-16			iLID 4-16		
Ct.Ar (mm^2^)	0.93	±	0.14	0.77	±	0.05*
Tt.Ar (mm^2^)	1.90	±	0.27	1.72	±	0.14
Ma.Ar (mm^2^)	0.97	±	0.19	0.94	±	0.10
J_o_ (mm^4^)	0.45	±	0.11	0.35	±	0.05
Ct.Th (mm)	0.20	±	0.03	0.17	±	0.01*
RCA	0.49	±	0.05	0.45	±	0.02
TMD (mg/cc)	1217	±	31	1184	±	49

Trabecular Bone Traits	Control 4-16			iLID 4-16		
BMD (mg/cc)	104	±	34	105	±	13
BV/TV (%)	19.4	±	4.5	22.5	±	1.6
TMD (mg/cc)	525	±	61	464	±	38
Tb.Th (mm)	0.054	±	0.004	0.050	±	0.001*
Tb.N (mm^-1^)	3.59	±	0.59	4.50	±	0.35*
Tb.Sp (mm)	0.23	±	0.05	0.17	±	0.02*

*Significantly different from control (ANOVA, p<0.05).

**Table 2:** Mean femoral histomorphometric data (± s.d) of cortical bone from male control (*n* = 7) and iLID mice (*n* = 9) at 16 weeks of age after injection with tamoxifen at 4 weeks.

		L. Pm			MAR			BFR/B. Pm			# Samples Lacking
		(%)			(µm/day)			(µm/day*100)			Double Labels
Periosteal	Control	32.4	±	10.2^a^	1.3	±	0.2	49.0	±	12.1	2
Surface	iLD	24.9	±	10.8a,b	1.2	±	0.4	35.6	±	20.1^a^	2
Endosteal	Control	54.1	±	16.7	1.2	±	0.1	69.3	±	23.5	1
Surface	iLID	44.7	±	9.8	1.1	±	0.3	50.3	±	15.1	0

a Significantly different from control endosteal,

b significantly different from iLID endosteal (ANOVA, *p*<0.05).

### Depletion of serum IGF-1 levels before and after peak bone acquisition affects cortical and trabecular bone architecture differently in the adult mouse

**Figure 5:**
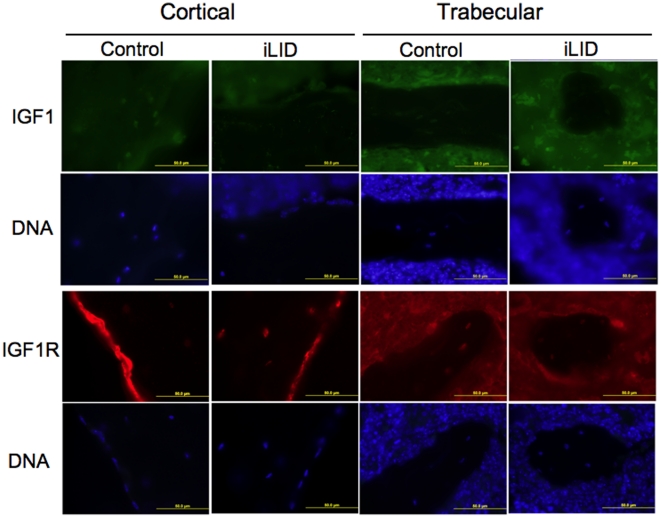
Representative immunofluroescene images of cortical and trabecular bone from 32 week control and iLID mice that were serum IGF-1 depleted at 16 weeks of age. Antibody staining for IGF-1 and the IGF-1R showed no differences when comparing control and iLID mice.

Additional groups of male control and iLID mice were injected once with tamoxifen (0.3 mg/mouse) at 8 or 16 weeks and were followed until 32 weeks (post-peak bone acquisition) (Figure 2A) to determine if a reduction in serum IGF-1 (Figure 2C) during the late growth phase (8 weeks) and at peak bone acquisition (16 weeks) would have different consequences during the adult growth phase (after peak bone acquisition). At 32 weeks body weights and body lengths were identical between iLID and control mice that had been injected with tamoxifen at 8 weeks (Figure 3B). Micro-CT data of cortical bone revealed significant reductions in Ct.Ar and Ct.Th of iLID mice at 32 weeks as compared to controls (Table 3, Figure 4B). Although histomorphometric analysis of cortical bone did not show significant differences between control and iLID mice at this age, likely due to the fact that bone formation has slowed tremendously by 32 weeks, mean values were all lower for iLID mice as compared to controls (Table 4). Trabecular bone architecture was not significantly different between 8 week injected iLID and control mice at 32 weeks of age (Table 3). For mice injected at 16 weeks, body weights of iLID mice also did not differ significantly from controls at 32 weeks (Figure 3B). Further, micro-CT analysis revealed that temporal depletion of serum IGF-1 at 16 weeks resulted in no significant alterations of cortical bone properties at 32 weeks of age (Table 3). However, we found significant changes in trabecular bone architecture where BMD, BV/TV, and Tb.N increased and Tb.Sp decreased in iLID mice (Table 3, Figure 4C). These findings were supported by histomorphometric data, indicating significant increases in mineralizing surface (MS) and bone formation rate (BFR) for iLID mice as compared to controls (Table 5). To determine if tissue-level expression of IGF-1 or the IGF-1R was altered in mice injected with tamoxifen at 16 weeks, we performed immunofluoresence on sections of 32 week cortical and trabecular bone from control and iLID mice and detected no differences between iLID and control mice (Figure 5).

**Table 3:** Mean cortical and trabecular bone traits values (± s.d) obtained from micro-CT measurements of male femora from control (*n* = 12) and iLID mice (*n* = 7) at 32 weeks of age after injection with tamoxifen at 8 weeks and male femora from control (*n* = 14) and iLID mice (*n* = 14) at 32 weeks of age after injection with tamoxifen at 16 weeks.

Cortical Bone Traits	Control 8-32			iLID 8-32			Control 16-32			iLID 16-32		
Tt.Ar (mm^2^)	1.87	±	0.22	1.77	±	0.11	1.93	±	0.13	1.90	±	0.20
Ct.Ar (mm^2^)	0.85	±	0.06	0.77	±	0.06*	0.88	±	0.05	0.85	±	0.07
Ma.Ar (mm^2^)	1.01	±	0.19	1.01	±	0.08	1.05	±	0.09	1.05	±	0.15
J_o_ (mm^4^)	0.41	±	0.07	0.36	±	0.04	0.44	±	0.05	0.42	±	0.08
Ct.Th (mm)	0.18	±	0.01	0.17	±	0.01*	0.19	±	0.01	0.18	±	0.01
RCA	0.46	±	0.05	0.43	±	0.03	0.46	±	0.02	0.45	±	0.03
TMD (mg/cc)	1330	±	45	1331	±	54	1338	±	30	1317	±	42

Trabecular Bone Traits	Control 8-32			iLID 8-32			Control 16-32			iLID 16-32		
BMD (mg/cc)	46	±	18	45	±	14	61	±	16	76	±	22*
BV/TV (%)	8.4	±	1.7	11.5	±	3.1	8.4	±	1.7	11.5	±	3.1*
TMD (mg/cc)	584	±	52	588	±	50	708	±	64	654	±	89
Tb.Th (mm)	0.029	±	0.004	0.029	±	0.003	0.034	±	0.004	0.033	±	0.004
Tb.N (mm^-1^)	2.66	±	0.98	2.64	±	0.81	2.49	±	0.44	3.48	±	0.96*
Tb.Sp (mm)	0.43	±	0.27	0.38	±	0.12	0.38	±	0.07	0.27	±	0.08*

*Significantly different from tamoxifen injection-matched control (ANOVA, *p*<0.05).

**Table 4:** Mean femoral histomorphometric data (± s.d) of cortical bone from male control (*n* = 10) and iLID mice (*n* = 6) at 32 weeks of age after injection with tamoxifen at 8 weeks.

		L. Pm			MAR			BFR/B. Pm			# Samples Lacking
		(%)			(µm/day)			(µm/day*100)			Double Labels
Periosteal	Control	39.6	±	8.5	0.7	±	0.2	27.2	±	9.6	0
Surface	iLD	32.0	±	18.0	0.5	±	0.1	20.3	±	9.8	1
Endosteal	Control	45.5	±	13.1	0.7	±	0.1	31.9	±	12.8	0
Surface	iLID	27.5	±	7.7	0.6	±	0.2	17.9	±	8.8	1

No significant differences were found.

**Table 5:** Mean femoral histomorphometric data (± s.d) of trabecular bone from male control (*n* = 6) and iLID mice (*n* = 7) at 32 weeks of age after injection with tamoxifen at 16 weeks.

	Control 16-32			iLID 16-32		
ES/BS	1.19	±	0.55	1.31	±	0.69
N.Ob/BS (#/mm)	10.74	±	2.81	12.59	±	1.33
N.Oc/BS (#/mm)	0.48	±	0.20	0.42	±	0.21
MS/BS (%)	38.80	±	3.51	66.08	±	8.59*
MAR (µm/day)	0.71	±	0.16	0.86	±	0.16
BFR/BS (µm/day)	0.28	±	0.07	0.57	±	0.11*

*Significantly different from control (ANOVA, *p*<0.05).

## Discussion

In humans, serum GH and IGF-1 decline gradually from ∼30 years of age [20], [21], while in mice serum IGF-1 levels remain fairly constant [22]. Mouse models with disrupted genes that encode components of the GH/IGF axis typically show developmental impairment of the skeletal system, which is carried on to adulthood [23], [24]. Moreover, tissue specific approaches, where genes of the GH/IGF system were ablated or overexpressed, were not able to distinguish the skeletal effect of IGF-1 during development and adulthood phases [25], [26]. In sharp contrast, the iLID model permits both temporal and spatial manipulation of the igf-1 gene, allowing us to induce igf-1 gene recombination specifically in the liver at different time points during growth, adulthood or aging.

In this study we present a novel application of the induced liver IGF-1 deficiency (iLID) mouse model for skeletal characterization. Our initial experiments were conducted to validate the iLID model for skeletal studies. We found that a single intraperitoneal injection of tamoxifen (0.3 mg) to iLID mice is sufficient to induce igf-1 gene recombination in the liver and consequently drop serum IGF-1 levels by ∼60%. In control mice, however, this dose of tamoxifen did not alter igf-1 gene expression in the liver, nor did it result in reductions in serum IGF-1 levels. Tamoxifen is a selective estrogen receptor modulator (SERM) that exhibits a potent anti-estrogen effect on mammary epithelium [27] and a partial agonistic effect on trabecular bone [28], [29], [30]. Thus, we assessed the consequences of tamoxifen injection in vivo on bone microarchitecture. We found that our selected tamoxifen dose (i.e., 0.3 mg) did not affect BV/TV in control mice at 4, 8, 16, or 32 weeks of age as no differences were found between tamoxifen and vehicle injected mice.

Using the iLID model we found that depletion of serum IGF-1 at 4 weeks of age (beginning of late growth phase) did not affect body weight or body length at 16 weeks. This is in contrast to the LID mice, which show constitutive reductions in serum IGF-1 from birth and thus significant reductions in body weight beginning at ∼12 weeks [24], [31]. We therefore conclude that maintenance of normal serum IGF-1 levels prior to puberty (before 4 weeks) enabled iLID mice to maintain body weight and length values that were identical to adult controls (16 weeks). Assessment of skeletal architecture showed that depletion of serum IGF-1 at the beginning of the late growth phase window (4 weeks of age) resulted in significant reductions in Tb.Th and Tb.Sp while Tb.N increased. In addition, there was a tendency towards increased BV/TV in iLID mice. Thus, depletion of IGF-1 during late growth results in changes to the trabecular architecture by adulthood. These changes may be due to elevations in GH levels that are found in iLID mice [24]. iLID mice depleted of IGF-1 at 4 weeks had significant reductions in Ct.Ar and Ct.Th as compared to control mice highlighting the importance of serum IGF-1 during the pubertal growth period. Given that significant growth is also achieved after puberty (8 weeks), we predicted that depletion of serum IGF-1 at 8 weeks should result in a measurable growth deficit at a later age. In support of this, injection of iLID mice at 8 weeks resulted in significant reductions in cortical bone properties (Ct.Ar, Ct.Th) by 32 weeks suggesting that reductions in serum IGF-1 in the middle of the late growth phase (8 weeks) can influence cortical bone properties in the older adult mouse. These findings are in agreement with previous findings regarding mouse development; although the greatest changes in cortical bone properties are seen before 16 weeks, new bone continues to be added more slowly well into adulthood [9], [13], [14]. Thus, although depletion of serum IGF-1 from birth or early post-natally (before 4 weeks) result in reductions in cortical bone accrual that exist throughout growth and into adulthood [32], reductions of serum IGF-1 levels during pubertal growth (late growth phase) are also important for establishing the cortical bone properties seen in later adulthood.

In the second part of our study we depleted IGF-1 at 16 weeks and assessed the skeletal response at 32 weeks of age. Mean Ma.Ar was not different between iLID and control mice at any age (regardless of the age of tamoxifen injection). In our previous study with the LID mice we show compensatory decreases in Ma.Ar by 32 weeks [24], likely due to inhibition of periosteal bone apposition. The absence of Ma.Ar changes in iLID mice suggests that compensatory marrow infilling occurs when a cumulative effect of reduced serum IGF-1 is present early post-natally and during puberty. In contrast to depletion at 4 weeks, depletion of serum IGF-1 at 16 weeks had no effect on cortical bone architecture assessed by 32 weeks of age, but did result in significant increases in trabecular BMD, BV/TV, and Tb.N. These results imply that serum IGF-1 depletion at maturity (after establishment of peak bone acquisition), does not affect cortical skeletal integrity and tissue mineral density (TMD) in the older adult mouse, but does alter trabecular architecture. Whether this trabecular enhancement can be maintained during aging (>52 weeks) is unknown. However, given the lack of a cortical bone phenotype in mice depleted of serum IGF-1 at 16 weeks and analyzed at 32 weeks, it is possible that the association of serum IGF-1 with BMD, although noted for peak bone mass in mice [33], may not hold during adulthood when bone mass gains slow and bone loss becomes more prevalent. Indeed, the correlations between serum IGF-1 and BMD noted in several human cohort studies of older individuals [34], [35], [36], [37], are likely not a direct cause and effect relationship.

One limitation to this study is that, although serum levels are reduced in the iLID model, compensatory changes in tissue level IGF-1 production may result. Robust measures of tissue-level IGF-1 are not easily obtained as any IGF-1 protein observed may still be a result of autocrine/paracrine or endocrine production. Further, such examinations would require knowledge of the specific time after serum IGF-1 depletion that corresponds to IGF-1 upregulation. However, we examined IGF-1 and IGF-1R expression in 32 week control and iLID animals by immunohistochemistry and found no differences. Further, in our iLID mice up-regulations in tissue IGF-1 are unlikely to be present or, if present, are insufficient given that impaired cortical skeletal development was observed by 16 weeks. Thus, we are confident that changes in tissue-level IGF-1 are not compensating for reductions in serum IGF-1.

This study and others have shown that heritable differences in circulating IGF-1 track with bone mass during growth (post-natal and during puberty) and are most evident during peak bone acquisition. Such differences may place certain individuals at an “at-risk starting point” for age-related bone loss. Our results of serum IGF-1-depleted adult mice suggest that reductions of IGF-1 in older adults have less impact on bone mass than reductions in serum IGF-1 during growth. We suggest that the relationships observed between IGF-1 and bone mass in the aging human are likely a “carry-over” from the IGF-1-dependent differences in bone morphology and composition that are established during early growth (post-natal to pubertal).
